# Gender Differences in Presentation, Management, and In-Hospital Outcomes for Patients with AMI in a Lower-Middle Income Country: Evidence from Egypt

**DOI:** 10.1371/journal.pone.0025904

**Published:** 2011-10-13

**Authors:** Neel M. Butala, Mayur M. Desai, Erika L. Linnander, Y. Rex Wong, Daoud G. Mikhail, Lesli S. Ott, John A. Spertus, Elizabeth H. Bradley, Ahmed Abdel Aaty, Alia Abdelfattah, Ayman Gamal, Hatem Kholeif, Mohamed El Baz, A. H. Allam, Harlan M. Krumholz

**Affiliations:** 1 Yale School of Medicine, New Haven, Connecticut, United States of America; 2 Yale School of Public Health, New Haven, Connecticut, United States of America; 3 Center for Outcomes Research and Evaluation, Yale–New Haven Hospital, New Haven, Connecticut, United States of America; 4 The Robert Wood Johnson Clinical Scholars Program, Department of Internal Medicine, Yale University School of Medicine, New Haven, Connecticut, United States of America; 5 Mid America Heart Institute, Kansas City, Missouri, United States of America; 6 Kansas City School of Medicine, University of Missouri, Kansas City, Missouri, United States of America; 7 Alexandria University, Alexandria, Egypt; 8 Cairo University, Cairo, Egypt; 9 Medinat Nasr Hospital, Cairo, Egypt; 10 Al-Azhar University, Cairo, Egypt; Kenya Medical Research Institute - Wellcome Trust Research Programme, Kenya

## Abstract

**Background:**

Many studies in high-income countries have investigated gender differences in the care and outcomes of patients hospitalized with acute myocardial infarction (AMI). However, little evidence exists on gender differences among patients with AMI in lower-middle-income countries, where the proportion deaths stemming from cardiovascular disease is projected to increase dramatically. This study examines gender differences in patients in the lower-middle-income country of Egypt to determine if female patients with AMI have a different presentation, management, or outcome compared with men.

**Methods and Findings:**

Using registry data collected over 18 months from 5 Egyptian hospitals, we considered 1204 patients (253 females, 951 males) with a confirmed diagnosis of AMI. We examined gender differences in initial presentation, clinical management, and in-hospital outcomes using t-tests and χ^2^ tests. Additionally, we explored gender differences in in-hospital death using multivariate logistic regression to adjust for age and other differences in initial presentation.

We found that women were older than men, had higher BMI, and were more likely to have hypertension, diabetes mellitus, dyslipidemia, heart failure, and atrial fibrillation. Women were less likely to receive aspirin upon admission (p<0.01) or aspirin or statins at discharge (p = 0.001 and p<0.05, respectively), although the magnitude of these differences was small. While unadjusted in-hospital mortality was significantly higher for women (OR: 2.10; 95% CI: 1.54 to 2.87), this difference did not persist in the fully adjusted model (OR: 1.18; 95% CI: 0.55 to 2.55).

**Conclusions:**

We found that female patients had a different profile than men at the time of presentation. Clinical management of men and women with AMI was similar, though there are small but significant differences in some areas. These gender differences did not translate into differences in in-hospital outcome, but highlight differences in quality of care and represent important opportunities for improvement.

## Introduction

Many studies in high-income countries have investigated gender differences in the care and outcomes of patients hospitalized with an acute myocardial infarction (AMI). The studies have indicated gender differences in the initial clinical presentation and the patterns of care, such as provision of evidence-based therapies [1], [2]. Evidence supporting gender differences in in-hospital mortality is mixed, though studies have shown that younger women with AMI compared with similarly aged men face a higher risk of dying in hospital [3].

In Egypt, cardiovascular disease has emerged the leading cause of death, and women are affected in large numbers [4]; however, little is known about the association of patient gender with patient care and outcomes in Egypt. Raising the specter of similar patterns within the Middle East, a regional study from six Arabian Peninsula countries found that women were less likely to receive certain treatments and had worse risk-adjusted in-hospital outcomes compared with men [5]. Nevertheless, these countries are distinctly different from Egypt, which has a much larger population, smaller percentage of expatriates, and a lower per capita income. Egypt is a lower-middle income country by World Bank standards [6], and the proportion of deaths stemming from cardiovascular disease is projected to increase dramatically in such countries with limited socioeconomic status [7].

Accordingly, we sought to examine gender differences in the presentation, management, and in-hospital outcomes of patients with AMI using registry data from 5 Egyptian hospitals. Our purpose was to determine if female patients with AMI have a different clinical presentation, treatment patterns, or outcome compared with men. Illuminating any disparities can help identify targets for quality improvement intervention and reveal particular patterns of care that may be prevalent in Egypt.

## Methods

### Ethics Statement

The Yale School of Medicine Human Investigations Committee (HIC) has determined that this study is not considered to be Human Subjects Research and is thus exempt from HIC review because the pre-existing data used in this study did not contain identifiable private information and were not collected by investigators through intervention or interaction with individuals. Written consent was not obtained because the data were analyzed anonymously.

The data in this study were collected as a part of a broader health system strengthening initiative. These data were not specifically collected for the current research project, and there is a documented mechanism in place that prevents the investigators from obtaining access to identifiers.

### Setting

This study uses data from an ongoing registry for patients with AMI in 5 Egyptian hospitals in Cairo and Alexandria, including 2 university-affiliated public hospitals, 1 public hospital affiliated with the Ministry of Health, 1 private hospital, and 1 university-affiliated hospital that has both public and private wings. These hospitals were purposefully selected to represent a diverse set of healthcare facilities in Egypt. The healthcare system in Egypt is highly pluralistic, with government sector providers funded solely by the Egyptian government, public sector providers funded by both the Egyptian government and other affiliated sources, and private sector providers [8]. Individuals with AMI who present to public or government hospitals generally do not have to pay out of pocket for services. Nearly all MOH tertiary care facilities, university hospitals, army and police hospitals, and large private hospitals (>100 beds) have CCUs. In these facilities, patients with AMI are generally admitted to the CCU, though specific criteria for admission to CCUs vary significantly by hospital.

### Participants and Study Design

Across the 5 hospitals in this study, a total of 1438 consecutive patients were admitted to the cardiac care unit (CCU) with suspected AMI over a period of 18 months between July 2009 and December 2010. Patients with a confirmed diagnosis of AMI, defined as a clinical suspicion of AMI with unequivocal EKG findings (ST-elevated myocardial infarction (STEMI) or STEMI-equivalent) or elevated serum levels of cardiac markers (troponin, CK-MB, creatinine) were included in the study; resulting in a total of 1204 patients meeting eligibility criteria.

Observations from the 238 patients who had incomplete discharge were excluded from analysis of discharge practices and in-hospital outcomes. These patients left the hospital for various reasons, such as leaving to obtain insurance approval or collect finances to make a payment for care. The percentage of patients with incomplete discharge status was similar by gender (19.8% for women vs. 20.0% for men). Observations from the 101 patients who were deceased were excluded from analysis of discharge practices.

### Data Collection

Data on admission, cardiac status on first medical contact, history and risk factors, medications, procedures, in-hospital clinical events, laboratory results, and discharge were collected during the hospitalization using a tool based on the American College of Cardiology NCDR ACTION-GWTG registry [9]. The NCDR ACTION-GWTG registry tool was modified through rapid cycles of change to ensure that it was culturally and logistically appropriate for Egypt, while allowing for comparisons with the United States [10]. To insure complete data collection, information pertaining to the index hospitalization was compiled on a data collection form used throughout the hospital stay by clinicians at each site. Information on past cardiac history, risk factors, and events before the index hospital admission was self-reported by patients to the clinicians completing the data collection form under the supervision of principal investigators in all sites.

Registry forms from the different hospital sites were collected during weekly site visits by research assistants from the coordinating center. The weekly site visits also served to share strategies for optimizing data collection among the sites and solicit feedback that could improve the process. Forms were screened for completeness on-site before being sent to a central office for data entry. Any registry forms received with unclear information were examined by a cardiologist before being sent back to the hospital sites for clarification, if necessary. Paper copies of all completed registry forms were kept in the central office after data had been entered for quality assurance. Monthly reports with site-specific and registry-wide summary statistics (not stratified by gender) were generated and circulated to each site in order to provide constant feedback to sites about data quality and performance on key indicators of healthcare quality.

### Measures

Variables examined included cardiac risk factors, cardiac history, cardiac status on admission, clinical management, and in-hospital outcomes. Cardiac risk factors included age, body mass index (BMI), smoking status, and diagnosis of hypertension, dyslipidemia, and diabetes mellitus according to Acute Coronary Syndromes Data Standards prior to arrival at first hospital [11]. Elements of prior cardiac history examined include heart failure, myocardial infarction, percutaneous coronary intervention (PCI), coronary artery bypass graft (CABG) surgery, and atrial fibrillation. Elements of cardiac status examined on admission included heart rate, blood pressure, and presence of STEMI on EKG between first medical contact and 24 hours after arrival at first hospital. Additionally we examined whether patients were transferred from another hospital.

Clinical management variables examined include use of aspirin in first 24 hours, anticoagulants (heparin, enoxaparin, or fondaparinux), GP IIb/IIIa inhibitor, utilization of echocardiogram, angiography, or reperfusion therapy in clinically eligible patients, fibrinolytic therapy in patients with STEMI, provision of smoking cessation counseling for smokers, scheduling of follow-up appointments, and administration of aspirin, beta blockers, and statins at discharge.

In-hospital outcomes that were prospectively collected included reinfarction according to criteria outlined above, cardiogenic shock, heart failure, cerebrovascular accident, suspected bleeding event, and in-hospital mortality. Many of these process and outcomes indicators are aligned with a range of “core quality measures” for AMI, as defined by the US Centers for Medicare & Medicaid Services (CMS) [12], including administration of aspirin at admission and discharge, smoking cessation counseling, and prescribing of beta blockers and statins at discharge.

### Statistical Analysis

The analysis was conducted in three steps. First, we examined gender differences in initial presentation upon arrival at the hospital. Specifically, we sought to determine whether male and female AMI patients differed with respect to cardiac risk factors, prior cardiac history, and cardiac status on admission; t-tests and χ2 tests were used for continuous and dichotomous variables, respectively. Similarly, we used t-tests and χ^2^ tests to examine differences in the clinical management and in-hospital outcomes between men and women. We also explored gender differences in in-hospital death using logistic regression modeling. Multivariable analysis was performed to adjust the sex effect for age and other differences in initial presentation; we fit the model using sequential adjustment for age, smoking status, hypertension, diabetes mellitus, prior CABG, prior heart failure, heart rate on admission, systolic blood pressure on admission, heart failure on admission, and ST-elevation. Our logistic regression modeling used generalized estimating equation techniques to account for the clustering of patients within hospitals [13]. All analyses were conducted using STATA 11.0 IC (StataCorp, College Station, TX).

## Results

Our analysis included 1204 patients (253 females, 951 males).

### Patient presenting characteristics

Women were significantly more likely to have risk factors for cardiovascular disease (Table 1). Women on average were older than men (p<0.001) and had a significantly higher BMI (p<0.001). In addition, they were more likely to have hypertension (69% vs. 47%; p<0.001), diabetes mellitus (58% vs. 40%; p<0.001), and dyslipidemia (31% vs. 18%; p<0.001). Men, however, were significantly more likely to be current or recent smokers (65% vs. 8%; p<0.001).

**Table 1:** Gender differences in initial presentation.

Variable	Sample	Men (% yes)	Women (% yes)	p-value from X^2^
N	1204	951	253	
*Cardiac Risk Factors*				
Mean Age^a^	1177	56.0(10.7)	61.0(11.3)	<0.001
Mean BMI^a^	1113	28.5(4.4)	31.3(5.2)	<0.001
Smoker	1179	604(65)	21(8)	<0.001
Hypertension	1194	445(47)	173(69)	<0.001
Dyslipidemia	957	134(18)	64(31)	<0.001
Diabetes Mellitus	1194	378(40)	147(58)	<0.001
*Prior Cardiac History*				
Prior MI	1185	167(18)	32(13)	0.061
Prior HF	1192	29(3)	26(10)	<0.001
Prior PCI	1188	118(13)	20(8)	0.042
Prior CABG	1186	31(3)	4(2)	0.149
Prior atrial fibrillation	1177	9(1)	11(4)	<0.001
*Cardiac Status on Admission*				
STEMI	1182	691(74)	143(58)	<0.001
Mean Heart Rate^a^	1170	86.1(21.9)	87.4(23.2)	0.441
Mean Systolic Blood Pressure^a^	1154	130.6(27.7)	134.1(30.9)	0.091
Heart Failure on Admission	1182	134(14)	51(21)	0.015
Transfer patient	987	171(22)	35(18)	0.202

a Mean (standard deviation) and p-values from t-test reported for continuous variables.

Prior cardiac history also differed significantly by gender. Women were more likely to have had prior heart failure (10% vs. 3%; p<0.001) and atrial fibrillation (4% vs. 1%; p<0.001), while men were more likely to have undergone prior PCI (13% vs. 8%; p<0.05). No significant difference in incidence of prior myocardial infarction or prior CABG was found.

Additionally, cardiac status on admission revealed significant gender differences. ST-elevated myocardial infarction was more common in men (74% vs. 58%; p<0.001), while heart failure upon admission was more common in women (21% vs. 14%; p<0.05). No significant difference in heart rate, systolic blood pressure, or transfer status was found.

### Clinical Management

Gender differences in the management of AMI were generally small though statistically significant for some processes of care (Table 2). Among patients without contraindications, women compared with men were slightly less likely to receive aspirin within 24 hours of admission (97% vs. 99%; p<0.01), anticoagulants during the hospital stay (95% vs. 98%; p<0.05), angiography during hospital stay (22% vs. 28%; p<0.05), aspirin at discharge (95% vs. 99%; p<0.01), or statins at discharge (92% vs. 96%; p<0.05). Additionally, women were more likely to be scheduled for a follow-up appointment (72% vs. 61%; p<0.05). In contrast, there were large gender differences in smoking cessation counseling, with women being less likely to receive smoking cessation counseling if they were smokers (73% vs. 92%; p<0.05). No significant gender differences were found in administration of GP IIb/IIIa inhibitors, use of echocardiography, reperfusion strategy, hospital duration, or administration of beta blockers at discharge.

**Table 2:** Gender differences in clinical management and in-hospital outcomes.

Variable	Sample	Men (% yes)	Women (% yes)	p-value from X^2^
**Clinical Management**				
**In-Hospital Medications**				
Aspirin in first 24 hours^a^	1195	936(99)	243(97)	0.004
Anticoagulants	1167	900(98)	232(95)	0.017
GP IIb/IIIa inhibitor	1162	107(12)	18(7)	0.058
**Diagnostics**				
Echocardiogram	1183	680(73)	188(76)	0.392
Angiography	1169	263(28)	53(22)	0.029
**Reperfusion**				
Reperfusion therapy if candidate	628	519(97)	89(96)	0.507
Thrombolytics if STEMI	602	457(90)	83(90)	0.876
***Discharge***				
Follow-up appointment scheduled	708	348(61)	101(72)	0.024
Smoking cessation counseling if smoker^a^	444	399(92)	8(73)	0.021
Aspirin at discharge_a_	835	664(99)	156(95)	0.001
Beta blocker at discharge^a^	818	591(89)	134(86)	0.232
Statins at discharge^a^	839	650(96)	150(92)	0.025
Hospital stay duration (days)^b^	813	5.6 (5.9)	5.8 (5.3)	0.682
**In-Hospital Outcomes**				
**Clinical events**				
Reinfarction	961	21(3)	10(5)	0.123
Cardiogenic shock	959	66(9)	26(13)	0.075
Heart failure	960	101(13)	31(15)	0.458
CVA	959	11(1)	0(0)	0.085
Major Bleed	945	32(4)	9(5)	0.859
**Mortality**	954	67(9)	34(17)	0.001

a Recognized by Centers for Medicare & Medicaid Services (CMS) as “Core Measures” for AMI for inpatient hospital quality.

b Mean(standard deviation) and p-value from t-test reported for continuous variables.

### In-hospital Complications

There were no significant gender differences in in-hospital clinical events, such as reinfarction, cardiogenic shock, heart failure, cerebrovascular accident, or suspected bleeding event (Table 2).

### In-hospital Mortality

The unadjusted in-hospital mortality rate for the entire sample was 10.6%, with a gender-specific mortality rate of 8.9% for males and 17.0% for females (p<0.01). Unadjusted logistic regression analysis indicated that the odds of in-hospital mortality was significantly higher for women compared with men (unadjusted OR: 2.10; 95% CI: 1.54 to 2.87) (Table 3). This difference persisted after adjustment for age (adjusted OR: 1.66; 95% CI: 1.14 to 2.42) but became non-significant after the addition of cardiac risk factors, including smoking, hypertension, and diabetes mellitus (adjusted OR: 1.26; 95% CI: 0.84 to 1.89). After subsequent adjustment for cardiac history, such as prior heart failure or prior CABG, the gender difference in in-hospital mortality was further attenuated (adjusted OR: 1.15; 95% CI: 0.74 to 1.81). Additional adjustment for cardiac status on admission had minimal effect on the point estimate for gender difference in in-hospital mortality but substantially widened the confidence interval (adjusted OR: 1.18; 95% CI: 0.55 to 2.55).

**Table 3:** Gender differences in in-hospital mortality.

	Men	Women	p-value*	Sample size
No. deceased/Total	67/754	34/200		
Mortality rate, %	8.89%	17.00%		
**Odds Ratio, OR (95% CI)**				
Unadjusted	1.00 (Ref.)	2.10 (1.54, 2.87)	<0.001	954
Adjusted for age	1.00 (Ref.)	1.66 (1.14, 2.42)	0.008	930
Adjusted for age and risk factors^b^	1.00 (Ref.)	1.26 (0.84, 1.89)	0.257	901
Adjusted for age, risk factors,^b^ and prior cardiac history	1.00 (Ref.)	1.15 (0.74, 1.81)	0.530	889
Adjusted for age, risk factors,^b^ prior cardiac history, and initial clinical presentation	1.00 (Ref.)	1.18 (0.55, 2.55)	0.670	829

a p-values based on adjustment for clustering of data within hospital.

b Risk factors adjusted for include smoking status, hypertension, diabetes mellitus.

## Discussion

Many studies in high-income countries have found gender differences in the presentation, care and outcomes of patients hospitalized with AMI [1]–[3]. This study is the first to investigate gender differences in the presentation, care and outcomes of AMI in Egypt, a country of lower-middle income status in the Middle East. We found that female patients have a significantly different clinical profile than men at the time of presentation. Additionally, clinical management of men and women with AMI was very similar, though there is indication that there are some areas where the differences are substantial. Specifically, women had small but significant deficiencies in the receipt of aspirin within the first 24 hours of presentation, anticoagulants or angiography during hospital stay, and aspirin or statins at discharge, but were substantially less likely to receive smoking cessation counseling and more likely to be scheduled for a follow-up appointment. However, these statistically significant gender differences in clinical management did not translate into differences in most in-hospital outcomes. While crude in-hospital mortality in female patients was significantly higher than mortality in male patients, this difference disappeared after adjusting for baseline characteristics.

In this study, 21% of patients with AMI in Egypt were female. This is a substantially lower percentage than reported in a large study conducted in developed countries throughout the world [14], but is comparable to the percentage of patients with AMI who were female in a study conducted in the Gulf region (21% in Egypt vs. 32% globally vs. 19% in Gulf region) [5]. Given that 44% of individuals in the Gulf registry were expatriates [15], the low percentage of female patients in Egypt and the Gulf prompt us to look at broader issues of access to care and care seeking behavior for women of this region.

Our findings of gender differences in Egyptian patients' initial clinical profile are congruent with findings from large national and global studies [3], [16]–[18]. A study using data from the Global Registry for Acute Coronary Events (GRACE) that examined 43,393 patients (14,180 women and 29,213 men) with acute coronary syndromes from 14 countries observed similar patterns. In the GRACE study, women with ACS were more likely to be older, have higher rates of hypertension, diabetes, and high cholesterol, and have prior heart failure compared with male patients, whereas men were more likely to smoke, have undergone previous PCI, and present with ST-elevated EKG findings upon admission [14]. It is interesting that gender differences in initial clinical profile among AMI patients in Egypt are similar to the gender differences found elsewhere, despite the younger average age and higher prevalence of risk factors, such as diabetes mellitus and smoking, overall in Egypt.

Consistent with other studies [19]–[21], we also found that female patients with AMI experienced small but significant differences in receipt of certain key services compared with male patients. One recent study by Jneid and colleagues examining gender differences in care processes among 78,254 patients with AMI in 420 US hospitals also found small but significant differences in the administration of aspirin within 24 hours [2]. However, Jneid et al. also documented the underuse of other evidence-based treatments and lower use of revascularization procedures for women. These additional findings regarding revascularization may not translate to Egypt, considering that resource constraints have led to limited adoption of catheterization as the primary reperfusion strategy in the participating hospitals. While the lack of gender differences in in-hospital outcomes following risk adjustment in our study is consistent with much of the literature [2], [20], [22], [23], the point estimate and confidence intervals in our study cannot exclude a clinically-important difference. Other studies have found that this risk-adjusted gender difference in mortality persists for younger women [1], [19], although age alone was not a major attenuating factor and such an effect was not pronounced in this study when repeated using stratified analysis.

The findings in Egypt are also comparable to findings from other countries in the region. The Gulf Registry of Acute Coronary Events (Gulf RACE) examined gender differences in 8,169 consecutive patients (74% men, 24% women) presenting with acute coronary syndromes from 6 adjacent Middle Eastern countries (Kuwait, Oman, United Arab Emirates, Yemen, and Bahrain) [5]. The study found that women were more likely to be older, have diabetes, hypertension, and dyslipidemia and were less likely to be smokers. The Gulf RACE study also noted a regional bias towards better clinical care for men, though the gender differences were slightly larger in magnitude than our study. While Gulf RACE did not find differences in administration of aspirin, statins, or anticoagulants in hospital, the study did find that women were significantly less likely to receive beta blockers (56% vs. 63%) and certain anti-platelet therapies (2% vs. 9%). Additionally, Gulf Race noted a significant difference in crude mortality rates among women and men with AMI (8.1% and 3.7%, respectively). However, these numbers are smaller in magnitude than the crude mortality rates noted in Egypt. In contrast to the findings on in-hospital outcomes in Egypt, the significant gender difference in in-hospital mortality in the Gulf RACE study persisted after adjustment for potential confounders (OR 1.76; 95% CI: 1.1 to 2.8), although the point estimate from the Gulf RACE study lies within the confidence interval from our fully adjusted model.

The gender differences in initial clinical profile in Egypt reflect similar differences found throughout the world [14]. It is notable that adjustment for this initial clinical profile results attenuated any significant gender differences in in-hospital mortality in our study. These differences in clinical profile may also help explain some of the differences in clinical care. For instance, the lower rate of administration of aspirin within the first 24 hours for female patients may be attributed to the fact that women may often present with atypical symptoms, and hospitals may not recognize a myocardial infarction in women initially [24], [25]. These differences in initial clinical presentation have implications for both provider education and sensitization to AMI in women.

The diminished rate of smoking cessation counseling in women compared with men represents a true gender disparity in quality of clinical care in Egypt. Nationally, Egyptian males are twice as likely to be smokers as females (40% men v. 18% women) [26]. The dramatically lower rate of smoking cessation counseling for women smokers in Egypt is striking, especially as smoking exacerbates the risk of acute coronary events for women in particular [27]. While only a minority of female patients with AMI report smoking, because smoking is perceived as a predominantly male activity in Egypt, cultural barriers or the risk of offending female patients may be preventing physicians from approaching the few women who do smoke. Further insights into the reasons for these differences and the creation of interventions to eradicate these differences in applying an important secondary prevention treatment need to be pursued.

This study extends the existing evidence base on gender differences among patients with AMI in lower-middle income countries, where the proportion deaths stemming from cardiovascular disease is projected to increase dramatically. One recent study from a coronary events registry in China, also a lower-middle income country, reported findings similar to ours, in terms of gender differences in clinical presentation, bias towards women in treatment, and lack of difference in in-hospital mortality [28]. However, data were only gathered from teaching hospitals, reflecting a higher level of medical care than is representative of health care delivery throughout China. The hospitals in our study were purposefully selected to represent a diverse set of healthcare facilities in Egypt. Additionally, one study from the lower-middle income country of Thailand found that women were older, had higher risk factors, and a higher risk for in-hospital complications [29]. However, it only examined differences among patients with ST-elevated AMI, which may introduce selection bias as our study found that female patients had significantly higher rates non-ST-elevated AMI compared with male patients. Finally, one of the countries in Gulf RACE, Yemen, can be classified as lower-middle income, but results on differences in gender were not reported independently for this country.

Nevertheless, this study's findings should be considered in light of its limitations. All patients were enrolled in the registry after admission to CCUs. While this allowed for standardization of inclusion criteria among disparate hospitals, the registry excludes patients with AMI who were admitted to other units or died before admission to the hospital and presents a possibility for selection bias. Because of a lack of consistent use of medical records across participating hospitals, validating the data after patient discharge was not generally possible. However, as providers were likely biased to record compliance with clinical guidelines, the observed differences in process measures of quality may be even greater in usual care, suggesting that we may have provided a conservative estimate of gender differences. Additionally, our relatively small sample size may have limited our statistical power in multivariate analysis due to the small number of female deaths in the final model. Finally, the study did not consider outcomes beyond in-hospital mortality, such a long-term survival, rehospitalization or quality of life, important targets for future studies.

In conclusion, this study focuses attention on opportunities for improvements in health care delivery in Egypt. Being cognizant of the drastically different initial clinical profile of women compared with men in Egypt can alert physicians to the greater absolute risk of in hospital mortality for women with AMI. Additionally, generally small but significant gender differences in guidelines-recommended care highlight potential deficiencies in quality of care and represent important opportunities for change. The evidence presented in this study on gender differences in patients with AMI can not only help improve quality of care in Egypt, but can also guide quality improvement in other lower-middle income countries, the areas of the world with the greatest current and future global burden of heart disease.
